# Colistin-Induced Acute Kidney Injury and the Effect on Survival in Patients with Multidrug-Resistant Gram-Negative Infections: Significance of Drug Doses Adjusted to Ideal Body Weight

**DOI:** 10.1155/2021/7795096

**Published:** 2021-12-20

**Authors:** Nittha Arrayasillapatorn, Palinee Promsen, Kittrawee Kritmetapak, Siriluck Anunnatsiri, Wijittra Chotmongkol, Sirirat Anutrakulchai

**Affiliations:** ^1^Phrakhon Chai Hospital, Prakhon Chai, Buri Ram, Thailand; ^2^Department of Internal Medicine, Kalasin Hospital, Mueang Kalasin, Kalasin, Thailand; ^3^Division of Nephrology, Department of Internal Medicine, Faculty of Medicine, Khon Kaen University, Khon Kaen, Thailand; ^4^Division of Infectious Diseases and Tropical Medicine, Department of Internal Medicine, Faculty of Medicine, Khon Kaen University, Khon Kaen, Thailand; ^5^Center of Excellence in Kidney Diseases, Srinagarind Hospital, Khon Kaen University, Khon Kaen, Thailand

## Abstract

**Background:**

Colistin is a lifesaving treatment for multidrug-resistant Gram-negative bacterial (MDR-GNB) infections along with its well-known nephrotoxicity. The controversy of colistin-induced acute kidney injury (AKI) on mortality is noted. This study aimed to determine the risk factors and impact of AKI on the survival and significance of colistin dosage.

**Methods:**

A retrospective cohort study was performed in adult patients who received intravenous colistin for MDR-GNB treatment between June 2015 and June 2017. Factors influencing colistin-induced AKI and survival were evaluated by Cox regression analysis. Cut-off levels of the colistin dose per ideal body weight (IBW) that significantly affected clinical outcomes were assessed with linearity trends and receiver operating characteristic analyses.

**Results:**

AKI occurred in 68.5% of 412 enrolled patients with an incidence rate of 10.6 per 100 patients-days and a median time was 6 (3–13) days. Stages I–III of AKI were 38.3, 24.5, and 37.2%. Factors associated with colistin-induced AKI were advanced age, high serum bilirubin, AKI presented before colistin administration, increased daily colistin doses per IBW, and concomitant use of nephrotoxic drugs. Colistin-induced AKI was related to mortality (HR 1.74, 95% CI 1.06–2.86, *p*=0.028). In the non-AKI before colistin usage subgroup, the total dose and total dose/IBW were >1,500–2,000 mg and 30–35 mg/kg to benefit mortality reduction but were <2,500–3,000 mg and 45–50 mg/kg for risk reduction of AKI. A daily colistin dose/IBW >4.5 mg/kg/day also increased the risk of AKI. In the AKI developed before colistin subgroup, the cut-off values of total colistin dose >1250–1350 mg and total dose/IBW >23.5–24 mg/kg demonstrated significant risks of AKI.

**Conclusion:**

The incidence of AKI after colistin administration was high and impacted mortality. Prevention and early correction of these related factors are mandatory. Careful use of colistin was also both beneficial in mortality and AKI reductions.

## 1. Introduction

Colistin is one of the polymyxin classes of antibiotics which was discovered in 1949 and was available for use in the 1960s [1]. Colistin use has resurged owing to the rising prevalence of infections caused by multidrug-resistant (MDR) and extensively drug-resistant (XDR) Gram-negative bacilli, especially *Pseudomonas aeruginosa, Acinetobacter baumannii, Klebsiella pneumoniae*, and carbapenem-resistant organisms [2]. Acute kidney injury (AKI) remains a treatment-limiting adverse effect of colistin, and physicians might hesitate to prescribe colistin in patients who have high risks for AKI. Prevalence of colistin-induced AKI was widely reported, ranging between 14.3% and 76.1% [3–6]. Median time to development of AKI ranged from 5 to 12 days [7–10]. Most patients who developed colistin-induced AKI had a renal recovery; however, some patients with severe AKI eventually required renal replacement therapy (RRT) [11, 12]. Noteworthy, most studies classified AKI according to RIFLE criteria (i.e., risk, injury, failure, loss of kidney function, and end-stage kidney disease [3, 13, 14]. Recently developed clinical practice guidelines from the kidney disease improving global outcomes (KDIGO) group provide uniform definition, staging, and severity of AKI which can detect AKI earlier and predict mortality more precisely than the previously used RIFLE criteria [15, 16].

Previous studies demonstrated that the risk factors of colistin-induced AKI were advanced age, hypoalbuminemia, hyperbilirubinemia, high daily doses of colistin, and concomitant use of other nephrotoxic agents [6, 8, 17, 18]. Colistin maintenance doses are mainly recommended according to renal function assessed by levels of creatinine clearance (CrCl) [19–21], and some suggestions consider drug doses varying with body weight (BW) of patients together with CrCl levels [22, 23]. Nevertheless, previous studies were concerned with the BW-adjusted doses in terms of microbiological responses and scarce information of BW and ideal BW- (IBW-) adjusted doses related to colistin-induced AKI and mortality [23–26]. Furthermore, there are still controversies over the recommended doses based on BW to optimize efficacy and nephrotoxicity and the impact of colistin-induced AKI on mortality due to different patient populations and multiple confounding factors [10, 13, 27]. Therefore, the purposes of this study were to determine the incidence, risk factors, and effect on mortality of colistin-induced AKI based on the recent KDIGOcriteria in a quite large sample of patients who received intravenous colistin mainly focusing on the associations between BW-adjusted colistin dosage and the occurrence of AKI and patient survival.

## 2. Methods

### 2.1. Study Design and Population

This retrospective cohort study was performed in the Srinagarind Hospital, KhonKaen University, KhonKaen, Thailand, between June 2015 and June 2017. Adult patients who were aged ≥18 years old and had received intravenous colistin for at least 48 hours for the treatment of multidrug-resistant Gram-negative bacteria (MDR-GNB) were included. MDR-GNB infection was defined as resistance to at least three of the five drug classes of antibiotics as follows: piperacillin/tazobactam, ceftazidime, or cefepime, carbapenems, aminoglycosides, and quinolones [28]. The exclusion criteria were chronic kidney disease (CKD), patients requiring renal replacement therapy (RRT, i.e., hemodialysis or peritoneal dialysis), and patients who had colistin-resistant Gram-negative bacilli infection. If patients received multiple courses of colistin treatment, only the first course of colistin treatment that met the inclusion or exclusion criteria was included.

The sample size calculation was based on the objective to evaluate factors associated with colistin-induced AKI by performing survival and Cox proportional hazard regression analyses. Data from previous studies showed varying hazard ratios around 1.1–1.8 and AKI prevalence about 30.8–39.9% as reported from Thai studies that were used as the reference values [4, 12, 13, 29]. A total sample size of 405 subjects was required for a power of 80% and a targeted significance level of 0.05.

The study protocol was reviewed and approved by the Institutional Ethics Review Board for Human Research of KhonKaen University (approval number HE601148) and was performed in accordance with the Helsinki Declaration. Since all data were obtained from available medical records, the informed consent requirement was waived.

### 2.2. Methods, Study Variables, and Definitions

Patient information was retrieved and reviewed from medical records in the hospital's computerized system. These data included demographic information, source of infection, biochemistry, microbiological laboratory data, colistin dosage and duration of treatment, acute kidney injury noted before and during colistin treatment, length of hospital stay, and results of treatment including microbiological response assessed by microbial eradication, i.e., no detection of the initial MDR-GNB at the end of colistin therapy, mortality at day 28, and overall mortality. In detail, patient characteristics included the following demographic and laboratory data: age, sex, BW, height, body mass index (BMI), IBW calculated by the Devine formula [30], underlying diseases, comorbidities and complications, Sequential Organ Failure Assessment (SOFA) score, presence of septic shock, parameters of severe sepsis, mechanical ventilator use, types of organisms, sites of infection, concomitant drugs (i.e., nonsteroidal anti-inflammatory drugs (NSAIDs), diuretics, vancomycin, aminoglycosides, amphotericin B, angiotensin-converting enzyme inhibitors (ACEI), angiotensin II receptor blockers (ARB), vasopressors, and radiological contrast media), laboratory data prior to colistin administration including hemoglobin, hematocrit, serum albumin, total bilirubin, blood urea nitrogen (BUN), creatinine (Cr), serum electrolytes, and baseline estimated glomerular filtration rate (eGFR) using the Chronic Kidney Disease Epidemiology Collaboration Creatinine Equation (CKD-EPI 2009) [31].

Colistin in a form of colistimethate sodium was intravenously administered at a dose calculated as a colistin-based activity with a loading dose of 300 mg and then followed by 150 mg intravenously every 12 hours if CrCl ≥50 ml/min calculated by using the Cockcroft-Gault equation. A maintenance dose was based on the hospital protocol which recommended adjustment of colistin doses according to the CrCl as suggested by previous pharmacokinetic studies [19, 21]. Occurrence and stages of AKI during colistin treatment were defined by changes of serially recorded serum creatinine and urine output as KDIGO criteria (Supplement Table (available here)) [14, 16]. Time of AKI development, changes of serum Cr, recovery of renal function, and requirement of RRT were recorded. Serum Cr and daily urine volumes were serially measured during colistin treatment, at the end of colistin treatment, before hospital discharge, and at the time of follow-up as out-patients. A recovery of AKI was defined as a return of Cr to non-AKI values and was evaluated up to 3 months after discharge from the hospital.

### 2.3. Outcome Measures

Main outcomes composed of an incidence of AKI during colistin treatment, a median time to AKI occurrence, and its associated factors were assessed by hazard ratios. Differences of characteristics between the AKI and the non-AKI groups were analyzed. The impact of AKI on patient survival was also evaluated by adjustment for other confounding factors. Both daily and total colistin doses adjusted with IBW were mainly focused factors of colistin-induced AKI and mortality.

### 2.4. Statistical Analyses

Statistical analyses were done by using STATA version 17.0. Continuous variables were expressed as mean ± standard deviation (SD) or median (interquartile range, IQR). Comparisons between the two groups used Student's *t*-test or Mann–Whitney *U* test as appropriate, and the paired Student's *t*-test was analyzed to identify the difference of before and after situations in the same group. Categorical data are shown as frequency (%) and tested for significance using the *χ*^2^ or Fisher exact test depending on the number of events. A univariate Cox regression analysis was used to investigate variables associated with AKI and death outcomes. Multiple variable analysis to determine the adjusted hazard ratios (HR) of related factors was evaluated by a full model of Cox proportional hazards regression analysis. Kaplan–Meier curves were constructed to show the median time of events and survival over time with the use of a two-sided stratified Log-rank test to compare the event groups and 95% confidence intervals (CI). A generalized estimating equation (GEE) was used to compare variables repeatedly measured between groups. For assessing the predictiveness of colistin dose adjusted with actual BW and IBW in forecasting the occurrence of AKI, linearity trends, receiver operating characteristic (ROC) curve analyses, and area under the ROC curve (AUC_ROC_) were determined. Levels of odds ratio (OR), sensitivity, specificity, positive predictive value (PPV), negative predictive value (NPV), the likelihood ratio for positive (LRP), and likelihood ratio for negative (LRN) are presented. All statistical analyses used the statistical significance value at *p* value < 0.05.

A priori target was set for an acceptable level of missing data as <5% and used a multiple imputation method for handling the missing data. These missing data were replaced with a set of predicted values imputed from other variables of the existing data, which contained the natural variability and uncertainty of the right values; then, multiple imputed data sets were combined and repeatedly analyzed to produce the final single overall analysis results.

## 3. Results

### 3.1. Baseline Characteristics of the Patients before Colistin Administration

A total of 576 patients received colistin therapy and 164 of them were excluded because of the duration of colistin use less than 48 hours (*n* = 21), underlying end-stage renal disease on RRT before receiving colistin (*n* = 118), younger than 18 years old (*n* = 8), and missing data (*n* = 17). Therefore, the data of 412 patients recruited for this study were assembled for analyses.  Table 1 presents the demographic data of the study patients at the beginning of colistin therapy. The mean age of subjects was 61.5 ± 18.9 years, 60.2% were male, and the mean actual BW and IBW were 55.8 ± 13.2 kg and 58.3 ± 9.5 kg. *Acinetobacter baumannii* (65.1%) and *Pseudomonas aeruginosa* (11.9%) were the most found organisms. Common underlying diseases of the patients were hypertension (HT, 32.5%), diabetes mellitus (DM, 21.8%), and coronary artery disease (CAD, 9.95%).

Twenty-one subjects (six percent) had septic shock and 60.9% were on respirators before colistin administration. Mean daily doses of colistin per actual BW and IBW were 5.28 ± 9.17 mg/kg and 4.75 ± 1.91 mg/kg. The median duration of colistin therapy was 10 (6–14) days, and the median length of stay was 38 (24–59) days.

### 3.2. Incidence Rate of Colistin-Induced AKI

**Figure 1:**
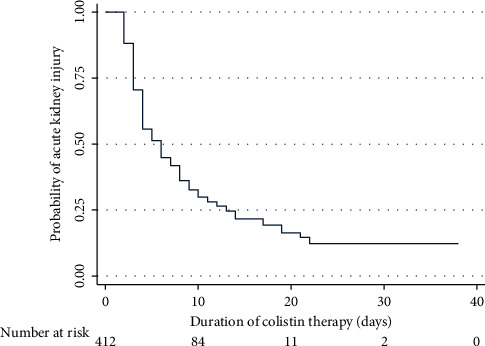
Kaplan–Meier curve demonstrates the probability of AKI occurrence during colistin treatment over time.

282 patients (68.5%) were defined as AKI complying with KDIGO criteria during colistin administration, accounting for an incidence rate of 10.6 per 100 patient-days with a median time to AKI of 6 (3–13) days as shown in Figure 1.  Table 1 reveals differences between the AKI and non-AKI groups. Significantly higher baseline characteristics found in the AKI group (a) were older, (b) had underlying diseases, e.g., DM, HT, CAD, and CKD, (c) had severe sepsis evaluated by levels of serum lactate, (d) had severe multiorgan failure assessed by SOFA scores, (e) had AKI before colistin administration, (f) used concomitant drugs affecting renal function, (g) had hyperbilirubinemia, and (h) had hypoalbuminemia.

### 3.3. Factors Related to Colistin-Induced AKI

The factors associated with AKI during colistin treatment analyzed with univariate analysis of Cox regression are shown in Table 2. After a full model of multivariate analysis to adjust for the significantly related independent variables, the factors related to AKI were (a) advanced age (HR 1.02, 95% CI1.01–1.02, *p* < 0.001), (b) high serum bilirubin (HR 1.03, 95% CI 1.01–1.06, *p*=0.006), (c) AKI presented before administration of colistin (HR 1.68, 95% CI 1.22–2.32, *p*=0.001) and higher risk if accompanied with oliguria (HR 2.91, 95% CI 1.46–5.80, *p*=0.002), (d) daily dose per IBW of colistin (HR 1.12, 95% CI 1.01–1.23, *p*=0.027), and (e) concomitant use of nephrotoxic drugs including vancomycin (HR 1.37, 95% CI 1.08–1.75, *p*=0.011), diuretics (HR 1.31, 95% CI1.01–1.70, *p*=0.041), ACEI (HR 2.30, 95% CI 1.06–4.99, *p*=0.036), and ARB (HR 7.72, 95% CI 1.04–57.1, *p*=0.045).

### 3.4. Characteristics, Severity, and Clinical Course of Colistin-Induced AKI

**Figure 2:**
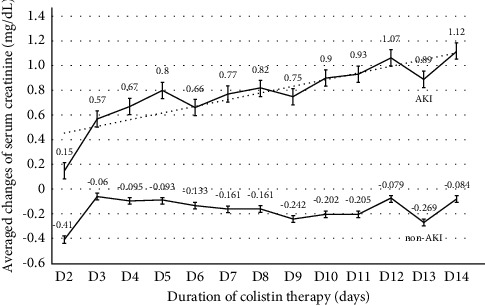
Means of serum creatinine changes from baseline levels in the AKI and non-AKI groups during 2 weeks of colistin treatment.

Table 3 shows the severity of AKI during colistin treatment. Of those 282 subjects, 108 (38.3%), 69 (24.5%), and 105 (37.2%) were classified as AKI stages 1, 2, and 3, of which 41 patients accounted for 14.5% of the AKI group who required RRT. The means of serum Cr changes from baseline levels during 2 weeks of colistin treatment are shown in Figure 2. The mean serum Cr levels of the non-AKI vs. AKI groups during colistin treatment were 1.11 ± 1.15 vs. 1.53 ± 1.23 (Day 2), 0.75 ± 0.41 vs. 2.03 ± 1.26 (Day 7), and 0.78 ± 0.58 vs. 2.18 ± 1.38 (Day 14); all *p* values <0.01. GEE showed the mean difference of serum Cr and eGFR adjusted with the baseline values, age, and sex during 14 days of colistin between the non-AKI and AKI groups was −0.83 (95% CI −0.65 to −1.01) mg/dL and 30.81 (95% CI 20.74–40.88) ml/min/1.73 m^2^. Individual comparisons of the baseline serum Cr before the start of colistin and the last serum Cr before discharge from the hospital or death revealed no significant change in the non-AKI group (0.97 ± 1.00 vs.1.04 ± 0.78, *p*=0.42) but significantly increased serum Cr and reduced eGFR in the colistin-induced AKI group (serum Cr; 1.25 ± 1.11vs.2.29 ± 1.65, *p* < 0.001 and eGFR 75.86 ± 37.90 vs.42.59 ± 30.70, *p* < 0.001).

In the AKI group, because 35 patients died during AKI progression and before the serial time of followed-up serum Cr, therefore, 247 cases were available for monitoring of renal recovery. Of those, 71 cases (28.7%) recovered from AKI with the median time of renal recovery of 30 (12.5–62) days. Their serum Cr means decreased from 2.14 ± 1.38 mg/dL (at colistin cessation) to 1.37 ± 0.76 mg/dL (at hospital discharge), 1.22 ± 0.61 mg/dL (at one-week follow-up), and 1.08 ± 0.96 mg/dL (at three-month follow-up). In the nonrecovery AKI group, serum Cr levels were 2.47 ± 1.36 mg/dL (at colistin cessation) vs. 2.65 ± 1.71 mg/dL at hospital discharge or time of death, *p*=0.04. 47.73% of them expired while serum Cr levels of live subjects at the hospital discharge and one week and three months later were 2.07 ± 1.54, 2.06 ± 1.74, and 1.67 ± 1.05 mg/dL compared with their baseline serum Cr 1.31 ± 1.12 mg/dL (all *p* values of every timepoint vs. baseline value <0.05).

### 3.5. Effect of AKI on Patient Survival

**Figure 3:**
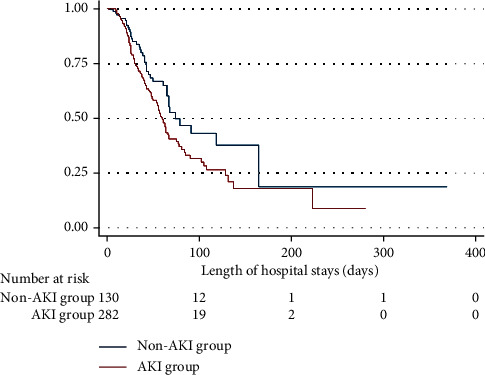
Kaplan–Meier curves of patient survival analysis compared between the colistin-induced AKI and non-AKI groups.

An overall mortality rate was 43% with an incidence rate of 0.91 per 100 patients-days and a median survival time of 64 days (95% CI 58–74 days). Furthermore, colistin-induced AKI affected mortality. The incidence of mortality in the AKI and non-AKI groups was 1.03 and 0.66 per 100 patients-days.  Figure 3 presents the Kaplan–Meier curves of both groups in which the median survival time in the AKI group was 60 days (95% CI 55–66 days) and the non-AKI group was 74 days (95% CI 65–164 days); *p* value assessed by Log-rank test = 0.02. Multivariate analysis by the Cox proportional hazard model was performed with adjustments for age, SOFA score, the severity of sepsis, serum albumin, type of organism, underlying diseases including CKD and malignancy, and dose of colistin. The results showed that colistin-induced AKI itself was an independent factor related to mortality (HR 1.57, 95% CI 1.09–2.28, *p*=0.016) especially the AKI that occurred in normal baseline renal function (HR 1.75, 95% CI 1.14–2.69, *p*=0.01) and the risk of mortality increased with severity of AKI (HR 1.81, 95% CI 1.04–3.15, *p*=0.036 for the RRT requirement group), and vice versa; the risk of mortality decreased in AKI whose renal function had recovered (HR 0.15, 95% CI 0.07–0.34, *p* < 0.001). The other factors related to increased mortality were (a) SOFA score (every increase of 1 score; HR 1.20, 95% CI 1.13–1.26, *p* < 0.001), (b) solid cancer (HR 2.30, 95% CI 1.56–3.38, *p* < 0.001), (c) hematologic malignancy (HR 2.62, 95% CI 1.57–4.37, *p* < 0.001), (d) serum albumin (every decrease of 1 g/dL; HR 1.63, 95% CI 1.23–2.16, *p*=0.001), and (e) septic shock (HR 1.98, 95% CI 1.29–3.03, *p*=0.002). Interestingly, every 1 mg/kg of the total dose of colistin adjusted with BW was associated with a reduction of mortality (HR 0.99, 95% CI 0.98–0.996, *p*=0.001).

### 3.6. Colistin Dosages and Clinical Outcomes in Subgroup Analyses

The multivariate analysis demonstrated a daily dose of colistin per IBW was one of the risk factors for overall colistin-induced AKI as shown in Table 1. Subgroup analyses to explore the significance of drug doses adjusted for BW or IBW as predictors of AKI in the two subgroups of colistin-induced AKI were performed: (1) AKI-naïve group, i.e., no AKI before colistin treatment (*n* = 182) and (2) AKI developed before colistin use and worsened during colistin administration (AKI before-worsening group, *n* = 100) (Table 4). Differences of drug dosages and clinical outcomes between the two subgroups of the non-AKI groups: (1) no AKI before colistin use (non-AKI before and after colistin group, *n* = 112) and (2) AKI before without AKI worsening during colistin treatment (AKI before nonworsening group, *n* = 18) are also shown in Table 4. There are no differences in loading dose percentage, loading dose/BW, and loading dose/IBW between the AKI and non-AKI groups. Because the given maintenance doses depended on the levels of CrCl, the patients who developed AKI before colistin administration received both daily doses and total doses less than the non-AKI before colistin group. Importantly, the AKI-naïve group were treated with significantly higher daily dose/IBW, total dose, total dose/BW, total dose/IBW, and longer duration than the non-AKI before and after colistin group. The poorest microbiological eradication was observed in the patients who developed AKI before colistin treatment. Overall mortality was the highest in the AKI before-worsening group (57%), and it was noted that the mortality rate of the AKI-naïve group (44%) was significantly higher than the non-AKI before and after colistin group (29.5%).

To clarify an independent risk factor of colistin dosage related to AKI and patient survival, the full models of multivariate analyses were performed by adjustment with other associated factors in the two subgroups; i.e., the first subgroup was the non-AKI before colistin (*n* = 294) further subdivided and compared between the AKI-naïve group (*n* = 182) and the non-AKI before and after colistin group (*n* = 112), and the second subgroup was the AKI before colistin (*n* = 118) further subdivided and compared between the AKI before-worsening group (*n* = 100) and AKI before nonworsening group (*n* = 18). In the non-AKI before colistin subgroup, every 1 mg/kg of daily colistin dose/IBW increased the risk of AKI with adjusted HR 1.23 (95% CI 1.05–1.44, *p*=0.009). The other significantly associated risks of colistin-induced AKI were an increase in age, lactic acid level, and total bilirubin, combination with vancomycin, ACEI, and diuretic use. Regarding survival, the occurrence of AKI during colistin was an increased risk of mortality (adjusted HR 1.68, 95% CI 1.07–2.66, *p*=0.025). The other related risks were an increased SOFA score, decreased serum albumin, and presence of solid and hematologic malignancies and septic shock. Importantly, the total dose of colistin/IBW revealed a protective effect on mortality (adjusted HR 0.99 for every increase of 1 mg/kg, 95% CI 0.98–0.996, *p*=0.002). In the AKI before colistin subgroup, the daily dose/IBW of colistin was not the risk related to worsening AKI, but preexisting CKD and receiving of ARB were the risks. In terms of survival studied by the multivariate analysis, worsening of AKI during colistin administration did not further increase the risk of mortality; however, the daily dose of colistin/IBW was associated with mortality (adjusted HR 1.53 for every increased dose 1 mg/kg, 95% CI 1.24–1.89, *p* < 0.001). Contradictory relations revealed that the total dose of colistin/IBW demonstrated a reduction of mortality with an adjusted HR of 0.98 for every increase of 1 mg/kg, 95% CI 0.966–0.999, *p*=0.043. The other mortality risks were male, underlying hematologic malignancy, increased levels of the SOFA score, and a decreased level of serum albumin.

The overall described results demonstrated that the high daily dose/IBW and total dose/IBW of colistin were related to the incidence of colistin-induced AKI, especially in patients with baseline normal renal function. Meanwhile, the total dose/IBW of colistin had a protective effect in mortality reduction. Therefore, linearity trends, AUCROC, and the likelihood ratio to determine cut-off values of daily dose/IBW and total dose/IBW of colistin showed a benefit in treatment and no harm regarding AKI complications as shown in Table 5. In the non-AKI before colistin subgroup, a total dose and a total dose/IBW should be more than 1,500–2,000 mg and 30–35 mg/kg to benefit mortality reduction but should be less than 2,500–3,000 mg and 45–50 mg/kg for risk reduction of AKI. A daily dose/IBW >4.5 mg/kg/day also demonstrated a risk of AKI, and if combined with vancomycin, a daily dose/IBW only >4 mg/kg/day increased the risk of AKI 3.86 times (OR 3.86, 95% CI 2.14–6.97) and AUC_ROC_ 0.63 with values of PPV 81.3%, NPV 47%, LRP 2.69 and LRN 0.70). In the AKI before colistin subgroup, only cut-off values of absolute total colistin dose (>1250–1350 mg) and total dose/IBW (>23.5–24 mg/kg) were demonstrated as the significant risks of AKI.

## 4. Discussion

The incidence of colistin-induced AKI in this study was high (68.5%) compared with previous data that reported ranges between 14.3% and 76.1% [3–5, 12, 29]. This variability could be attributed to varying definitions of AKI because most previous studies defined AKI by using RIFLE criteria, while this study detected AKI based on the newer KDIGO criteria, which can earlier detect AKI especially with the definition of serum Cr increased 0.3 mg/dL from the baseline value within 48 hours (Supplementary Materials). Few recent studies reported the incidence of colistin-induced AKI defined with KDIGO criteria in adult patients of 29–68.9% varying with severity of infection, organ failure, comorbidities, and underlying diseases [6, 32–34] comparable with the current study. The median time to AKI development revealed in the present study was 6 days, conforming to previous studies [3, 27, 35]. The postulated mechanism of colistin-induced nephrotoxicity is due to the d-aminobutyric and fatty acid components of the drug, which increase renal tubular cell membrane permeability resulting in cell lysis and acute tubular necrosis [1]. In an animal model, histopathological examination in the colistin-treated group showed renal tubular injury evidenced by dilatation of tubules, degeneration and necrosis of epithelial cells, and cast formation [36]. There were also case reports of acute interstitial nephritis due to hypersensitivity to this antibiotic in which renal pathologies were characterized by interstitial edema, heavy interstitial infiltration of eosinophils, plasma cells, and lymphocytes, with occasional polymorphonuclear leukocyte [37, 38].

Independent factors associated with colistin-induced AKI in the present study were advanced age, AKI at admission especially with oliguria, concomitant use of nephrotoxic drugs (vancomycin, ACEI, ARB, diuretics), a high daily dose of colistin per IBW, and high serum bilirubin. Previous studies revealed no association of loading doses with the incidence of colistin-induced AKI [6, 9, 39, 40], consistent with the present study (HR 1.09, 95% CI 0.74–1.60, *p*=0.66). Several studies, however, have shown a strong relationship between the colistin daily dosage and AKI risk [3, 9, 11, 34]. Colistin-induced nephrotoxicity by itself is dose-dependent and related to the length of exposure [9]. In a previous retrospective study, Pogue et al. found that the nephrotoxicity usually occurred within the first week of treatment, and there was a 30% increased risk of toxicity in patients receiving colistin of 3 to 4.9 mg/kg/day (based on IBW) and up to 69% when giving colistin ≥5 mg/kg/day [3]. The present study demonstrated similar findings in which a high daily dose of colistin per IBW increased colistin-induced nephrotoxicity. In general, there are 2 patterns of CMS dosing guideline recommendations, either fixed dosing or a weight-based dosing regimen, both adjusted for renal function [1]. In the KKU hospital, using fixed dosing of colistin may increase the chance of deleterious exposure to the higher dose of colistin treatment, especially in low body weight patients.

Although a high dose of colistin was associated with nephrotoxicity, an inadequate dose was also related to mortality. Analyses of cut-off colistin dosages in this study with evidence of significant OR and mild-moderate predictors by AUC_ROC_ suggested that, in the non-AKI before colistin subgroup, the total dose 1500–3000 mg and total dose/IBW 30–50 mg/kg and, in the AKI before colistin subgroup, the total colistin dose <1350 mg and total dose/IBW <24 mg/kg would be in the range of acceptable risk and benefit. This study supported the concept that even with normal baseline renal function, the physician should be aware of an appropriate colistin dose, and for meticulous colistin use, both daily dose and the total dose adjusted for CrCl and IBW should be thoroughly considered [22–24, 26, 41–43]. In 2019, the international consensus guidelines for the optimum therapeutic use of polymyxins were developed by multiple global organizations of physicians and pharmacists, which demonstrated the importance of drug dosing and AKI prevention [26].

Concurrent uses of other potentially nephrotoxic drugs with colistin have been reported as augmentation risks of AKI; for example, vancomycin can induce kidney injury via proinflammatory oxidation, mitochondrial dysfunction, and cellular apoptosis [44]. Whether treatment with combined vancomycin and colistin increases the risk of AKI is still controversial [6, 11, 34, 45] depending on related factors such as the studied population, severity of illness, drug dosage, and duration of the combination. The findings of multivariate analysis in this study supported the augmented risk of AKI by vancomycin. Vancomycin is usually used for the treatment of methicillin-resistant *Staphylococcus aureus*, and some studies suggested the synergistic effect of vancomycin plus colistin in the clearance of MDR-*Acinetobacter baumannii* [46, 47]. Katip and Oberdorfer, however, demonstrated no apparent clinical benefit nor mortality reduction of vancomycin add-on colistin in the treatment of carbapenem-resistant *Acinetobacter baumannii* [45]. Diuretics, ACEI, and ARB can affect renal hemodynamics, especially in critically ill patients who are susceptible to poor renal perfusion. Therefore, to improve patient safety in health care systems, medications should be carefully reviewed before using colistin and other potentially nephrotoxic drugs being avoided or used with great caution.

Consistent with previous data [48, 49], preexisting AKI was also a significant risk factor of colistin-induced nephrotoxicity in this study. High peak and trough colistin plasma levels at a steady state have been found in patients with baseline renal impairment despite dose titration corresponding to renal function [50]. Severe sepsis and multiorgan dysfunction impair glomerular filtration and intrarenal autoregulation, which may enhance colistin nephrotoxicity. No increased AKI hazard risk of septic shock was noted, however, and the presence of hyperlactatemia only trended toward statistical significance for AKI development in the current study. Hyperbilirubinemia was found to be a risk factor for colistin-induced AKI. In patients with hyperbilirubinemia due to liver disease, increased angiotensin II and nitric oxide resulted in renal hypoperfusion and synergistically impaired renal function [51–53].

This current study revealed that AKI development during colistin treatment was an independent factor for mortality, which confirmed some previous studies [27, 50, 54]. Furthermore, the severity of AKI had an impact on patient outcomes as oliguria and RRT requirements increased the risk of death, while in patients who had a renal recovery, the mortality was lower. Therefore, detection of AKI at the early stage is essential to slow disease progression. Moreover, ensuring an environment that best allows for renal recoveries, such as the maintenance of adequate hemodynamics, appropriate types and doses of antibiotic usage, avoidance of nephrotoxic agents, and providing good nutrition, is mandatory.

The limitation of this study is that, with the retrospective design, there may be some missing information that could lead to a selection bias and is unaccounted for by the confounder. Because the patients in this study had medium asthenic bodies, i.e., an average IBW around 55–60 kg, interpretations with a much larger body size should be used with caution. Among severely ill patients, data on body weight had to be collected from the recent data record or patients' relatives or by estimation, which may have affected the calculated renal function, IBW, and colistin dosing treatment. Some patients with prolonged hospitalization developed other conditions, e.g., hypoalbuminemia, hypovolemia, superinfection, or shock that could affect the progression of renal function deterioration. The strength of the study was enough to study subjects with multiple infectious characteristics; therefore, factors related to AKI and death outcome were adequately analyzed and can be referred to as external generalizations.

Further studies are needed to explore whether manipulation of these modifying factors can reduce the risk of AKI. New strategies to prevent this high rate of colistin-induced AKI by lowering doses of intravenous colistin, for example, therapeutic drug monitoring, combined with other beneficial antibiotics and inhalation of colistin are encouraged.

## 5. Conclusions

In conclusion, due to the high incidence of colistin-induced AKI with a high mortality rate, physicians should use colistin with increased awareness especially in high-risk patients who are elderly, receiving other concurrent nephrotoxic drugs, have high bilirubin levels, and present with preexisting AKI. Appropriate colistin dose was also both beneficial in mortality and AKI reductions.

## Figures and Tables

**Table 1:** Demographic data, baseline characteristics of patients with and without AKI during colistin treatment.

Characteristics	Total (*n* = 412)	AKI (*n* = 282)	Non-AKI (*n* = 130)	*p* value
Age (years)^a^	61.5 ± 18.9	65.6 ± 17.1	52.6 ± 19.7	<0.001
Male, *n* (%)	247 (60.2)	172 (61.0)	75 (57.7)	0.646
Actual BW (kg)^a^	55.8 ± 13.2	55.5 ± 12.5	56.5 ± 14.6	0.456
IBW (kg)^a^	58.4 ± 9.47	57.8 ± 9.46	59.4 ± 9.42	0.111
BMI (kg/m^2^)^a^	21.5 ± 4.78	21.5 ± 4.61	21.5 ± 5.14	0.892
Underlying disease, *n* (%)				
Hypertension	134 (32.5)	104 (36.9)	30 (23.1)	0.005
CKD	96 (23.3)	79 (28.0)	17 (13.1)	0.001
Diabetes mellitus	90 (21.8)	73 (25.9)	17 (13.1)	0.003
Coronary artery disease	41 (9.95)	38 (13.5)	3 (2.31)	<0.001
On respirator, *n* (%)	251 (60.9)	173 (61.7)	78 (60.0)	0.794
SOFA score^b^	4.5 (2–7)	5 (3–7)	4 (2–6)	0.004
Septic shock, *n* (%)	89 (21.6)	67 (23.8)	22 (16.9)	0.117
Concomitant drugs, *n* (%)				
Vancomycin	152 (36.9)	121 (42.9)	31 (23.9)	<0.001
Diuretics	110 (26.7)	87 (30.9)	23 (17.7)	0.005
Vasopressors	102 (24.8)	79 (28.0)	23 (17.7)	0.024
Radiological contrast media	23 (5.58)	18 (6.38)	5 (3.85)	0.297
Amphotericin B	19 (4.61)	15 (5.32)	4 (3.08)	0.313
NSAIDS	6 (1.46)	4 (1.42)	2 (1.54)	1.000
Aminoglycoside	9 (2.18)	7 (2.48)	2 (1.54)	0.726
ACEI	8 (1.94)	7 (2.48)	1 (0.77)	0.444
ARB	1 (0.24)	1 (0.35)	0 (0)	1.000
Number of concomitant drugs, *n* (%)				<0.001
1 concomitant drug	147 (35.7)	102 (36.2)	45 (34.6)	
2 concomitant drugs	86 (20.9)	74 (26.2)	12 (9.23)	
3 concomitant drugs	29 (7.04)	23 (8.16)	6 (4.62)	
4 concomitant drugs	6 (1.46)	5 (1.77)	1 (0.77)	
Baseline laboratory				
Hemoglobin (g/dL)^a^	9.39 ± 1.69	9.29 ± 1.67	9.59 ± 1.73	0.095
Serum albumin (g/dL)^a^	2.64 ± 0.69	2.60 ± 0.71	2.74 ± 0.63	0.046
Total bilirubin (mg/dL)^b^	2.17 ± 5.04	2.52 ± 5.86	1.42 ± 2.28	0.040
Mean serum creatinine (mg/dL)^a^	1.16 ± 1.08	1.25 ± 1.11	0.97 ± 1.00	0.015
Median serum creatine (mg/dL)^b^	0.8 (0.6–1.3)	0.9 (0.6–1.5)	0.67 (0.5–1.1)	0.015
eGFR (mL/min/1.73 m^2^)^a^	82.5 ± 40.2	75.9 ± 37.9	96.8 ± 41.6	<0.001
Severe sepsis, *n* (%)				
Lactate >18 mg/dL	75 (18.2)	60 (21.3)	15 (11.5)	0.017
Baseline eGFR <60 ml/min/1.73m^2^, *n* (%)	127 (30.8)	100 (35.5)	27 (20.8)	0.005
AKI before colistin administration	31 (7.52)	20 (7.10)	11 (8.46)	
AKI on top CKD	87 (21.1)	80 (28.4)	7 (5.38)	
CKD without AKI	9 (2.18)	0 (0)	9 (6.92)	
Oliguria ≥2 hours before colistin, *n* (%)	10 (2.43)	10 (3.55)	0 (0)	0.030
Site of infection, *n* (%)				
Pneumonia	288 (69.9)	196 (69.5)	92 (70.8)	0.795
Urinary tract infection	36 (8.74)	27 (9.57)	9 (6.92)	0.376
Septicemia	35 (8.50)	23 (8.16)	12 (9.23)	0.716
Soft tissue infection	75 (18.2)	52 (18.4)	23 (17.7)	0.855
Type of microorganism, *n* (%)				
*Acinetobacter baumannii* (MDR)	268 (65.1)	186 (66.0)	82 (63.1)	0.569
*Pseudomonas aeruginosa* (MDR)	49 (11.9)	35 (12.4)	14 (10.8)	0.632
*Klebsiella pneumonia* (MDR)	19 (4.61)	14 (4.96)	5 (3.85)	0.802

*Note.* ^a^Mean ± SD, SD; standard deviation, ^b^median (interquartile range: IQR). AKI: acute kidney injury, IBW: ideal body weight, BMI: body mass index, CKD: chronic kidney disease, SOFA: sequential organ failure assessment, ACEI: angiotensin-converting enzyme inhibitors, ARB: angiotensin II receptor blockers, NSAIDs: nonsteroidal anti-inflammatory drugs, GFR: glomerular filtration rate, and MDR: multidrug-resistant.

**Table 2:** Univariate and multivariate analyses to identify the factors associated with colistin-induced AKI.

Variable	Crude HR (95% CI)	*p* value	Adjusted HR (95% CI)	*p* value
Age (every 1-year increase)	1.02 (1.02–1.03)	<0.001	1.02 (1.01–1.02)	<0.001
CKD (with/without)	1.87 (1.43–2.43)	<0.001	0.95 (0.65–1.39)	0.806
Vancomycin (with/without)	1.38 (1.09–1.74)	0.008	1.37 (1.08–1.75)	0.011
ACEI (with/without)	1.93 (0.91–4.11)	0.087	2.30 (1.06–4.99)	0.036
ARB (with/without)	8.56 (1.18–62.0)	0.034	7.72 (1.04–57.1)	0.045
Diuretics (with/without)	1.47 (1.14–1.89)	0.003	1.31 (1.01–1.70)	0.041
Baseline total bilirubin level (every increase of 1 mg/dL)	1.03 (1.01–1.05)	0.003	1.03 (1.01–1.06)	0.006
Septic shock (with/without)	0.77 (0.59–1.02)	0.065	1.21 (0.89–1.63)	0.224
Lactate >18 mg/dL (with/without)	1.75 (1.31–2.34)	<0.001	1.34 (0.98–1.82)	0.065
AKI before colistin administration (with/without)	2.22 (1.73–2.85)	<0.001	1.68 (1.22–2.32)	0.001
AKI with oliguria ≥2 hours before colistin administration	4.04 (2.13–7.67)	<0.001	2.91 (1.46–5.80)	0.002
Daily dose/IBW of colistin (every increase of 1 mg/kg)	0.92 (0.85–0.99)	0.037	1.12 (1.01–1.23)	0.027
Total dose/IBW of colistin (every increase of 1 mg/kg)	0.994 (0.990–0.998)	0.002	0.996 (0.992–1.001)	0.132

*Note.* AKI: acute kidney injury, HR: hazard ratio, CI: confidence interval, CKD: chronic kidney disease, ACEI: angiotensin-converting enzyme inhibitor, ARB: angiotensin receptor blockade, and IBW: ideal body weight.

**Table 3:** Characteristics of acute kidney injury after administration of colistin.

Characteristics of AKI	AKI group (*n* = 282)
Stage of AKI based on KDIGO criteria, *n* (%)	
Stage 1	108 (38.29)
Stage 2	69 (24.46)
Stage 3	105 (37.23)
Median time after colistin administration (days)	6 (3–13)
Median time after admission (days)	19 (12–28)
Renal replacement therapy, *n* (%)	41/282 (14.5)
Renal recovery, *n* (%)	71/247 (28.7)
Median time of renal recovery (days)	30 (12.5–62)

*Note*. AKI: acute kidney injury and KDIGO: kidney disease improving global outcomes.

**Table 4:** Comparison of colistin dosage, length of hospital stay, and death outcomes between AKI and non-AKI groups.

Characteristic	Colistin-induced AKI				No colistin-induced AKI			
	Total AKI (*n* = 282)	AKI-naïve (*n* = 182)	AKI before colistin and worsening during colistin Rx (*n* = 100)	*p* value	Total non-AKI (*n* = 130)	No AKI before and during colistin treatment (*n* = 112)	AKI before colistin but no worsening during colistin Rx (*n* = 18)	*p* value
Body weight (kg)	55.5 ± 12.5	54.8 ± 12.6	56.8 ± 12.3	0.20	56.5 ± 14.6	56.8 ± 15.2	54.8 ± 10.1	0.59
Ideal body weight (kg)	57.8 ± 9.46	57.6 ± 9.06	58.2 ± 10.2	0.63	59.4 ± 9.42	59.8 ± 9.69	57.3 ± 7.44	0.30
Serum creatinine before treatment with colistin (mg/dL)	1.23 ± 1.09^*∗*^^,#,$^	0.68 ± 0.24^*∗*^^,$^	2.23 ± 1.32^*∗*^^,#^	<0.001	0.97 ± 1.00	0.76 ± 0.55	2.26 ± 1.88	<0.001
Serum creatinine at time off colistin (mg/dL)	2.37 ± 1.42^*∗*^^,#^	1.94 ± 1.20^*∗*^^,#^	3.16 ± 1.45^*∗*^^,#,$^	<0.001	0.92 ± 0.88	0.74 ± 0.38	2.02 ± 1.85	<0.001
Colistin treatment and dosing								
Loading of colistin, *n* (%)	253 (89.7)	159 (87.4)	94 (94.0)	0.08	120 (92.3)	102 (91.1)	18 (100)	0.36
Loading dose (mg)	292 ± 33.1	297 ± 20.5	284 ± 46.3^*∗*^^,#^	0.003	296 ± 24.6	296 ± 22.3	292 ± 35.4	0.45
Loading dose/BW (mg/kg)	5.52 ± 1.31	5.70 ± 1.34	5.20 ± 1.22	0.003	5.52 ± 1.28	5.53 ± 1.30	5.48 ± 1.19	0.88
Loading dose/IBW (mg/kg)	5.22 ± 1.07	5.32 ± 0.96	5.03 ± 1.21	0.036	5.11 ± 0.95	5.10 ± 0.95	5.17 ± 0.93	0.78
Daily dose (mg/day)^a^	253 ± 80.2^*∗*^^,#,$^	294 ± 37.1^*∗*^^,$^	178 ± 83.3^*∗*^^,#^	<0.001	272 ± 66.6	286 ± 51.9	185 ± 81.9	<0.001
Daily dose/BW (mg/kg/day)^a^	4.77 ± 1.85^#,$^	5.63 ± 1.41^*∗*^^,$^	3.20 ± 1.52^*∗*^^,#^	<0.001	5.03 ± 1.61	5.30 ± 1.49	3.35 ± 1.35	<0.001
Daily dose/IBW (mg/kg/day)^a^	4.50 ± 1.61^#,$^	5.23 ± 1.05^*∗*^^,#,$^	3.17 ± 1.63^*∗*^^,#^	<0.001	4.66 ± 1.31	4.89 ± 1.16	3.22 ± 1.32	<0.001
Total dose of colistin (mg)^a^	2,852 ± 2,009^$^	3,529 ± 2,095^*∗*^^,#,$^	1,627 ± 1,038^*∗*^^,#^	<0.001	2,506 ± 1,662	2,693 ± 1,662	1,342 ± 1,121	0.001
Total dose of colistin/BW (mg/kg)^a^	53.3 ± 36.6^$^	66.6 ± 37.3^*∗*^^,#,$^	29.2 ± 18.8^*∗*^^,#^	<0.001	46.4 ± 33.8	49.9 ± 34.3	24.9 ± 19.3	0.003
Total dose of colistin/IBW (mg/kg)^a^	50.8 ± 39.0^*∗*^^, $^	63.2 ± 41.8^*∗*^^,#,$^	28.4 ± 18.0^*∗*^^,#^	<0.001	43.2 ± 31.0	46.3 ± 31.5	23.9 ± 19.3	0.004
Duration of colistin treatment (days)^a^	10 (7–14)^*∗*^^,#,$^	10 (7–14)^*∗*^^,#,$^	7.5 (5.5–14)^$^	0.001	7 (5–14)	7 (5–14)	4.5 (3–14)	0.08
Microbiological eradication, *n* (%)	180 (63.8)	122 (67.0)	58 (58.0)^#^	0.19	92 (70.8)	84 (75.0)	8 (44.4)	0.042
Length of stay (days)^b^	40 (25–59)	40 (25–60)	39.5 (26–58)	0.85	35 (20–61)	34.5 (20–61)	36 (24–68)	0.76
Death within days 28 of admission, *n* (%)	57 (20.2)^#^	36 (19.8)	21 (21.0)	0.81	16 (12.3)	13 (11.6)	3 (16.7)	0.47
Mortality rate, *n* (%)	137 (48.6)^*∗*^^,#^	80 (44.0)^*∗*^^,#^	57 (57.0)^*∗*^^,#^	0.036	40 (30.8)	33 (29.5)	7 (38.9)	0.42

*Note.* ^a^Mean ± SD, SD; standard deviation, ^b^median (interquartile range: IQR), AKI: acute kidney injury, BW: body weight, and IBW: ideal body weight. *p* values <0.05; ^*∗*^vs. total non-AKI group, ^#^vs. no AKI before and during colistin treatment group, and ^$^vs. AKI before colistin but no worsening during colistin treatment group.

**Table 5:** Predictors of total and daily colistin dosages as the risks of acute kidney injury and benefactors of mortality reduction.

	OR (95% CI)	Sensitivity (%)	Specificity (%)	PPV (%)	NPV (%)	LRP	LRN
Risks for colistin-induced AKI							
Non-AKI before colistin (*n* = 294)							
Daily colistin dose/IBW (AUC_ROC_ 0.57)							
>4.0 mg/kg/day	2.59 (1.25–5.37)	92.3	17.9	64.5	58.8	1.12	0.43
>4.5 mg/kg/day	1.75 (1.05–2.90)	75.1	36.6	65.7	47.7	1.19	0.68
>5.0 mg/kg/day	1.48 (0.93–2.38)	58.0	51.8	66.0	43.3	1.20	0.81
Daily colistin dose/BW (AUC_ROC_ 0.55)							
>5.5 mg/kg/day	1.60 (1.00–2.59)	50.0	61.1	67.9	43.1	1.30	0.81
Total colistin dose (AUC_ROC_ 0.63)							
>1,500 mg	2.49 (1.41–4.40)	85.1	30.4	66.4	55.7	1.22	0.49
>2,000 mg	2.01 (1.19–3.40)	79.0	34.8	66.2	50.6	1.21	0.60
>2,500 mg	3.04 (1.87–4.95)	66.3	60.7	73.2	52.7	1.69	0.56
>3,000 mg	1.95 (1.20–3.19)	48.1	67.9	70.7	44.7	1.50	0.77
Total colistin dose/IBW (AUC_ROC_ 0.65)							
>45 mg/kg	3.29 (2.02–5.37)	67.2	61.6	73.8	53.9	1.75	0.53
>50 mg/kg	2.68 (1.65–4.37)	58.9	65.2	73.1	49.7	1.69	0.63
AKI before colistin (*n* = 118)							
Total colistin dose (AUC_ROC_ 0.62)							
>1,000 mg	3.21 (1.18–8.76)	72.0	55.6	90.0	26.3	1.62	0.50
>1,200 mg	2.92 (1.07–7.97)	65.0	61.1	90.3	23.9	1.67	0.57
>1,250 mg	3.74 (1.28–10.9)	59.0	72.2	92.2	24.1	2.12	0.57
>1,350 mg	3.59 (1.23–10.4)	58.0	72.2	92.1	23.6	2.09	0.58
Total colistin dose/IBW (AUC_ROC_ 0.61)							
>20.0 mg/kg	3.66 (1.30–10.2)	64.6	66.7	91.4	25.5	1.94	0.53
>22.5 mg/kg	3.83 (1.31–11.1)	59.6	72.2	92.2	24.5	2.15	0.56
>23.5 mg/kg	3.12 (1.07–9.04)	54.5	72.2	91.5	22.4	1.96	0.63
>24.0 mg/kg	3.00 (1.03–8.68)	53.5	72.2	91.4	22.0	1.93	0.64
Predictors for improved survival							
Non-AKI before colistin (*n* = 294)							
Total colistin dose (AUC_ROC_ 0.57)							
>1,500 mg	2.20 (1.16–4.17)	89.0	21.4	64.7	54.5	1.13	0.52
>2,000 mg	2.08 (1.23–3.53)	79.6	34.8	66.4	51.3	1.22	0.59
Total colistin dose/IBW (AUC_ROC_ 0.57)							
>25 mg/kg	2.21 (1.19–4.11)	87.8	23.4	65.2	54.2	1.15	0.52
>30 mg/kg	1.84 (1.07–3.17)	80.7	30.6	65.5	49.3	1.16	0.63
>35 mg/kg	2.20 (1.33–3.66)	75.7	41.4	67.8	51.1	1.29	0.59

*Note.* OR: odd ratio, PPV: positive predictive value, NPV: negative predictive value, LRP: likelihood ratio for positive results, and LRN: likelihood ratio for negative results.

## Data Availability

The data analyzed and presented in this study are available from the corresponding author upon request.

## Supplementary Materials

Supplementary Materials Table S1: definitions and stages of AKI according to the RIFLE and KDIGO criteria.
