# Supplementary material for: Colistin-Induced Acute Kidney Injury and the Effect on Survival in Patients with Multidrug-Resistant Gram-Negative Infections: Significance of Drug Doses Adjusted to Ideal Body Weight
Source: Int J Nephrol. 2021 Dec 20;2021:7795096. doi: 10.1155/2021/7795096 (PMC8712152; doi:10.1155/2021/7795096)
Supplement: Supplementary Materials — Table S1: definitions and stages of AKI according to the RIFLE and KDIGO criteria. [file 7795096.f1.docx]

**Table S1** Definitions and Stages of AKI according to the RIFLE and KDIGO criteria [14,16]

**RIFLE criteria [14]**

| **Classification** | **GFR criteria** | **Urine output criteria** |
| --- | --- | --- |
| **R**isk | Increased S_Cr_ x 1.5 or GFR decrease > 25 % | <0.5 ml/kg/hour x 6 hours |
| **I**njury | Increased S_Cr_ x 2 or GFR decreased > 50 % | <0.5 ml/kg/hour x 12 hours |
| **F**ailure | Increased S_Cr_ x 3 or GFR decreased > 75 % or S_Cr_ ≥ 4.0 mg/dL with an acute rise ≥ 0.5 mg/dL | < 0.3 ml/kg/hour x 24 hours or anuria x 12 hours |
| **L**oss | Complete loss of renal function > 4 weeks | |
| **E**SKD | End Stage Kidney Disease (> 3 months) | |

**KDIGO criteria [16]**

| **Definition of AKI** | **Stage** | **Serum creatinine** | **Urine output** |
| --- | --- | --- | --- |
| Increase in S_Cr_ ≥ 0.3 mg/dL within 48 hours  or S_Cr_ ≥ 1.5 time of baseline over 7 days  or urine volume < 0.5 ml/kg/hour for 6 hours | 1 | Increased S_Cr_ 1.5-1.9 times from baseline over 7 days or a ≥ 0.3 mg/dL absolute increase over 48 hours | <0.5 ml/kg/hour for 6-12 hours |
|  | 2 | Increased S_Cr_ 2.0-2.9 times from baseline | <0.5 ml/kg/hour for >12 hours |
|  | 3 | Increased S_Cr_ 3 times from baseline  or S_Cr_ >4.0 mg/dL or initiation of RRT | < 0.3 ml/kg/hour for >24 hours or anuria for >12 hours |
